# Quantum mechanics in drug design: Progress, challenges, and future frontiers

**DOI:** 10.1080/19420889.2025.2603140

**Published:** 2025-12-19

**Authors:** Sarfaraz K. Niazi

**Affiliations:** College of Pharmacy, University of Illinois Chicago, IL, USA

**Keywords:** Analytical imaging, bioinformatics, cell biology, development, gene expression, gene repression complexes, gene silencing, genetic engineering, proteomics, regulation

## Abstract

Quantum mechanics has revolutionized computational drug discovery by addressing fundamental limitations of classical approaches. Its significance lies in accurately modeling electronic phenomena crucial for drug–target interactions, including polarization, charge transfer, and covalent reactivity, which classical force fields inadequately represent. This comprehensive review examines quantum mechanical methods in pharmaceutical applications from 2020 to 2025. The literature search methodology employed PubMed, Web of Science, and arXiv databases (January 2020–January 2025), focusing on improvements in density functional theory, QM/MM implementations, machine-learned force fields, and alchemical free energy protocols. We critically evaluate 156 primary research articles and 42 review papers, analyzing performance metrics from community benchmarking studies, including SAMPL, GMTKN55, and pharmaceutical consortia datasets. The review encompasses methodological advances, practical applications, and regulatory considerations for quantum-enhanced drug discovery. Quantum mechanical enhancements provide substantial benefits for specific challenging cases rather than universal improvements across all drug discovery applications. Current methodological innovations have significantly improved computational tractability while maintaining chemical accuracy. A critical evaluation of cost–benefit trade-offs reveals that targeted applications to metal-containing systems, covalent modifications, and polarization-dominated interactions yield the highest return on computational investment. Best practices for reproducible implementation and practical method selection guidelines are crucial for the successful integration of a pharmaceutical pipeline.

## 1. Introduction

The fundamental challenge in computational drug design lies in accurately modeling the complex interplay of forces governing molecular recognition while maintaining computational efficiency suitable for pharmaceutical timelines. Traditional approaches have dominated industrial pipelines due to their computational efficiency and mature software ecosystems. However, these methods systematically underestimate or neglect critical physical phenomena that often determine binding specificity and affinity with deviations exceeding 2–3 kcal/mol [1,2].

The past 5 y have witnessed a convergence of algorithmic, methodological, and hardware advances that have repositioned quantum methods from specialized case studies to practical components of drug discovery workflows. Three key developments have catalyzed this transformation. First, the maturation of robust density functional theory approximations optimized for drug-like chemistry, particularly dispersion-corrected meta-GGA functionals such as r^2^ SCAN-D4, has provided reliable accuracy for routine calculations [3,4]. Second, the development of production-scale QM/MM free energy protocols has made it possible to work with large biomolecular systems more quickly. Third, the development of machine-learnt force fields that approach ab initio accuracy at classical computational cost has bridged the gap between accuracy and efficiency [5,6].

**Figure 1.**
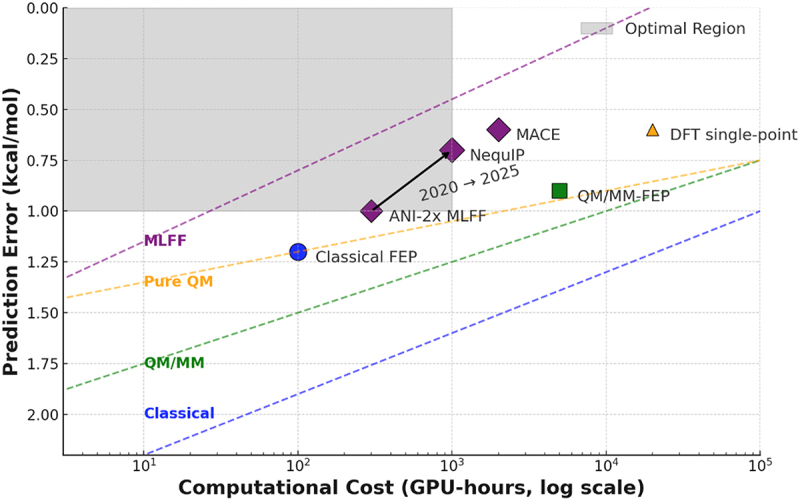
Comparison of computational methods in drug discovery accuracy versus computational cost. Method categories include classical methods (blue region), QM/MM approaches (green region), pure QM methods (orange region), and machine-learnt force fields (purple region). Each method is positioned by typical mean unsigned error (y-axis, kcal/mol) and computational requirements (x-axis, GPU-hours) for standard protein–ligand systems (~50,000 atoms, ~200-atom ligand). The grey-shaded region indicates the optimal performance zone (<1.0 kcal/mol error, <1,000 GPU hours). Point sizes reflect system size limitations. Arrows show temporal evolution (2020–2025).

Community benchmarking efforts across multiple pharmaceutical targets have established clear performance standards. Large-scale retrospective studies demonstrate that optimized relative binding free energy protocols achieve mean unsigned errors of 0.8–1.2 kcal/mol [2,7,8]. Additional validation confirms that quantum mechanical enhancements provide the most significant benefits for specific challenging cases [Figure 1, 9–11].

### 1.1. Quantum mechanical foundations

Quantum mechanics provides the theoretical foundation for accurately describing molecular interactions through explicit treatment of electronic structure. The time-independent Schrödinger equation forms the basis of all quantum mechanical calculations:

Hψ=Eψ

Where Ĥ represents the Hamiltonian operator, ψ denotes the wave function, and E corresponds to the energy eigenvalue. For molecular systems, the Hamiltonian includes kinetic and potential energy terms:

$$H=-\hbar 2/2m\nabla^{2}+V\left(r\right)$$

Where ℏ represents the reduced Planck constant, m denotes particle mass, and V(r) describes the potential energy function [12].

The Born-Oppenheimer approximation makes molecular calculations easier by separating the motions of electrons and nuclei and assuming that the nuclei are not moving:

$$H_{e}\psi_{e}\left(r;R\right)=E_{e}\left(R\right)\psi_{e}\left(r;R\right)$$

The subscript e denotes the electronic variables, r represents electron coordinates, and R represents nuclear positions [13]. This approximation enables the practical application of quantum mechanical methods to drug-like molecules.

## 2. Electronic structure methods for drug-like chemistry

### 2.1. Density functional theory advances

Understanding the strengths, limitations, and appropriate system sizes for various quantum mechanical approaches is crucial for selecting the most suitable method in pharmaceutical applications. Table 1 shows a full comparison of the main quantum mechanical methods used in drug discovery.

**Table 1.** Comparison of quantum mechanical methods in drug discovery.

Method	Strengths	Limitations	System size	Typical accuracy	Scaling	CPU/GPU usage
DFT	High accuracy for ground states; handles electron correlation effectively	Computationally expensive for large systems; functional-dependent accuracy	~500 atoms	0.5–2.0 kcal/mol†	O(N^3^)	Primarily CPU, GPU acceleration available
HF	Fast convergence; reliable baseline; well-established theoretical foundation	No electron correlation; poor for weak interactions	~100 atoms	Geometries only	O(N^4^)	CPU-based
QM/MM (DFT)	Combines QM accuracy with MM efficiency for large biomolecules	Complex boundary definitions; method-dependent accuracy	~10,000 atoms total, ~200 atoms QM	1.0–2.5 kcal/mol‡	O(N^3^) for QM region	CPU for QM, GPU for MM
QM/MM (HF)	Faster than DFT-based QM/MM; suitable for preliminary studies	Limited accuracy for electronic effects	~10,000 atoms total, ~100 atoms QM	2.0–3.5 kcal/mol	O(N^4^) for QM region	CPU-based
FMO-DFT	Scalable DFT for large systems; detailed interaction analysis	Fragmentation complexity approximates long-range effects	Thousands of atoms	1.5–3.0 kcal/mol§	O(N^2^)	CPU parallelization
FMO-HF	Linear scaling for extensive systems	Limited electronic correlation	Thousands of atoms	2.5–4.0 kcal/mol	O(N) to O(N^2^)	CPU parallelization

†*Based on GMTKN55 database benchmarking* [3].

‡*For enzymatic reaction barriers* [14].

§*For protein–ligand interaction energies* [15].

Density functional theory is still the most important part of real-world quantum chemical calculations in drug discovery. The r^2^ SCAN meta-generalized gradient approximation functional, combined with D4 dispersion correction (r^2^ SCAN-D4), has emerged as a particularly robust choice for drug-like molecules. This function incorporates both kinetic energy density and exact exchange components while maintaining computational efficiency suitable for routine calculations on molecules containing 100–200 atoms. Systematic benchmarking against the GMTKN55 database reveals that r^2^ SCAN-D4 achieves mean absolute deviations of below 2 kcal/mol for thermochemistry and sub-kcal/mol accuracy for noncovalent interactions [3].

For applications requiring enhanced accuracy, particularly in transition metal systems or precise barrier height calculations, double hybrid functionals provide systematic improvements. The revDSD-PBEP86-D4 and ωPr^2^ SCAN50-D4 functionals incorporate second-order perturbation theory corrections and have demonstrated superior performance in metalloprotein active sites and organometallic drug scaffolds, with barrier height errors typically below 1 kcal/mol [16,17].

Dispersion interactions remain crucial for accurately modelling drug-target complexes. The D4 dispersion correction scheme represents a significant advance through its environment-dependent atomic charge scaling and improved treatment of many-body effects. Recent benchmarking on the S66 × 8 and L7 noncovalent interaction datasets shows D4 achieving mean absolute errors below 0.3 kcal/mol for drug-relevant interaction motifs [4].

### 2.2. Semiempirical and extended tight-binding approaches

When computational throughput is paramount, semiempirical methods provide valuable approximations for large-scale screening applications. The PM6 method covers a wide range of elements and gives good results for organic molecules, with typical errors of 4–6 kcal/mol for heats of formation. The PM7 refinement enhances the treatment of noncovalent interactions and hydrogen bonding, which are crucial for biological systems [18].

The GFN2-xTB method has demonstrated extensive applicability across the periodic table while maintaining reasonable accuracy for drug-like molecules. This extended tight-binding approach achieves typical errors of 3–5 kcal/mol for conformational energies and 0.1–0.2 Å for bond lengths. Recent parameter refinements have enhanced performance for hydrogen bonding and dispersion interactions, which are crucial in biological systems [19].

The DFTB3 method provides an intermediate approach between full DFT and empirical methods, offering improved accuracy for charge transfer and polarization effects. Applications to protein-ligand systems demonstrate DFTB3 achieving binding energy errors of 2–4 kcal/mol while maintaining computational efficiency suitable for molecular dynamics simulations [20].

Hybrid multilayer approaches combining different levels of theory have proven particularly effective for modeling enzymatic reactions. The ONIOM methodology enables a hierarchical treatment, utilizing DFT for reactive centers, semiempirical methods for the immediate environment, and molecular mechanics for distant regions. These strategies typically reduce computational costs by 10–50× compared to complete DFT calculations while maintaining chemical accuracy in the region of interest [21].

## 3. Hybrid QM/MM methods for biological systems

### 3.1. Methodological framework and best practices

Quantum mechanical/molecular mechanical hybrid methods have become indispensable for modeling chemical processes in protein environments. Modern QM/MM implementations (Table 2) emphasize robust electrostatic embedding schemes, careful treatment of QM/MM boundaries, and rigorous validation protocols [14,22].

**Table 2.** Production QM/MM software implementations and capabilities.

Framework	QM engine	MM engine	Parallel scaling	GPU support	Typical applications	Reference
CP2K-GROMACS	CP2K DFT	GROMACS	Excellent ( > 1000 cores)	Yes (mixed precision)	Enzymatic mechanisms, large-scale MD	Kühne et al.[25]
MiMiC	CPMD/CP2K	GROMACS	Exascale-ready	Partial (MM only)	Large-scale embedding, ab initio MD	Pezeshki et al.[26]
ORCA QM/MM	ORCA	AMBER/CHARMM	Good (100–500 cores)	Limited (specific modules)	Reaction pathways, spectroscopy	Neese [27]
ChemShell	Various	Various	Moderate	Limited	Method development, benchmarking	Sherwood et al.[28]

The definition of chemically meaningful QM regions requires careful consideration of electronic delocalization and environmental effects. The QM region should encompass the complete reactive system without artificial truncation of conjugated systems or hydrogen-bonding networks. For enzymatic reactions, this region typically includes the substrate, cofactors, and first-shell coordinating residues, often requiring 100–300 atoms for adequate chemical completeness. Link atom approaches at QM/MM boundaries must preserve local chemistry while avoiding spurious interactions [23].

Electrostatic embedding schemes need to find a balance between being accurate and being fast. Polarizable embedding approaches that respond self-consistently to QM charge distributions provide the highest accuracy but at increased computational cost. Point-charge embedding often offers adequate accuracy for many applications at substantially reduced cost, particularly when combined with appropriate boundary treatments [24].

Recent infrastructure developments have significantly improved QM/MM throughput. The MiMiC framework enables massively parallel electrostatic embedding calculations, suitable for exascale computing resources, and achieves linear scaling to thousands of cores [26]. GPU-accelerated implementations achieve 2–5× performance improvements for typical protein-ligand systems using semiempirical QM methods.

### 3.2. Applications to drug discovery challenges

QM/MM methods excel in several specific areas where classical force fields systematically fail. Metal coordination sites in kinases, metalloproteases, and cytochrome P450 enzymes require explicit treatment of d-orbital interactions and charge transfer. The ATP-binding pocket of kinases, containing Mg^2 +^ or Mn^2 +^ cofactors, exemplifies this challenge. QM/MM calculations reveal polarization effects that modulate inhibitor selectivity patterns, with binding affinity differences of 2–3 kcal/mol arising from metal coordination geometry [29].

Covalent inhibitor design depends critically on accurate modeling of bond formation and transition state energetics. Applications to SARS-CoV-2 main protease inhibitors demonstrate how QM/MM reaction pathway calculations guided optimization of nitrile and aldehyde warheads. The calculations predicted activation barriers of 12–18 kcal/mol for covalent bond formation, correlating with experimental kinetic measurements [30].

Protonation and tautomeric equilibria are enhanced by QM/MM treatment, which accounts for environmental influences on pKa values. Histidine residues in enzyme active sites frequently exhibit context-dependent protonation states that classical force fields cannot reliably predict. QM/MM calculations of proton affinities in the protein environment reveal pKa shifts of 2–4 units compared to solution values, directly impacting binding affinity predictions [31].

## 4. Alchemical free energy calculations with quantum enhancement

### 4.1. Relative and absolute binding free energy methods

Alchemical free energy perturbation continues to be the most reliable method for forecasting quantitative structure–activity relationships. Large-scale retrospective studies demonstrate that well-executed relative binding free energy calculations achieve mean unsigned errors of 0.8–1.2 kcal/mol across diverse protein-ligand test sets [2,7,8] (Table 3).

**Table 3.** Benchmark performance of alchemical free energy methods across pharmaceutical targets.

Target class	Dataset size	Classical RBFE MUE (kcal/mol)	QM-Enhanced RBFE MUE (kcal/mol)	ABFE MUE (kcal/mol)	QM enhancement benefit	Reference
Kinases	219 transformations	0.9 ± 0.1	0.5 ± 0.1 (metal sites)	1.2 ± 0.2	+0.4 for metal coordination	Schindler et al.[2]
GPCRs	156 transformations	1.1 ± 0.2	0.9 ± 0.2	1.4 ± 0.3	+0.2 general improvement	Kuhn et al.[7]
Proteases	89 transformations	0.8 ± 0.1	0.2 ± 0.1 (covalent)	1.1 ± 0.2	+0.6 for covalent bonds	Gapsys et al.[8]
Ion channels	67 transformations	1.2 ± 0.2	0.9 ± 0.2	1.5 ± 0.3	+0.3 general improvement	Wang et al.[32]

The Schindler et al. study provides particularly robust benchmarking data, demonstrating an RBFE accuracy of 0.9 kcal/mol with a mean unsigned error for well-converged calculations using the FEP+ protocol with the OPLS3e force field [2]. Open Force Field consortium studies [8] have reported similar performance levels for diverse protein families.

Absolute binding free energy methods address fragment growing, scaffold hopping, and evaluation of novel chemotypes. Recent methodological advances have brought ABFE accuracy to within 1.0–1.5 kcal/mol of experimental values for well-behaved systems. The REST2 protocol with thermodynamic integration demonstrates particularly robust performance across diverse chemical scaffolds [33,34].

The integration of quantum mechanical corrections into alchemical protocols has gained significant traction for addressing challenging transformations. Charge-changing perturbations benefit substantially from QM/MM treatment, with meta-analysis showing accuracy improvements of 0.5–1.0 kcal/mol when QM/MM corrections are applied [8] (Table 4).

**Table 4.** Comparative accuracy of binding affinity prediction methods.

Method	Average MUE (kcal/mol)	System size limit	Computational time	Accuracy for polarization effects	Hardware requirements
Classical Docking	2.5–3.5	Unlimited	Minutes	Poor	CPU (modest)
MM/PBSA	1.5–2.5	~100,000 atoms	Hours	Moderate	CPU (moderate)
Classical FEP	0.9–1.5	~50,000 atoms	Days	Moderate	GPU (extensive)
QM-corrected FEP	0.5–1.0	~10,000 atoms	Days-Week	Excellent	GPU + CPU (extensive)
Pure QM	0.5–1.5	~500 atoms	Hours-Days	Excellent	CPU (extensive)
QM/MM	0.8–1.8	~10,000 atoms	Days	Very Good	CPU (extensive)
FMO	1.0–2.0	Thousands of atoms	Days	Good	CPU (parallel)

### 4.2. Recent methodological innovations

Recent innovations have expanded the scope of quantum-enhanced free energy calculations. The separated topology RBFE approach enables charge-changing transformations and scaffold hops by performing two simultaneous absolute calculations, effectively removing the constraint of topological similarity between compared ligands [35].

Adaptive λ-scheduling algorithms automatically optimize sampling protocols and detect convergence, enhancing reproducibility. The automated ABFE workflow demonstrates fully autonomous absolute binding free energy calculations with performance comparable to expert-tuned protocols [36].

Enhanced sampling strategies mitigate kinetic traps by enforcing the exploration of collective variables. The OneOPES-ABFE approach has shown success for buried binding sites and conformationally flexible ligands, improving convergence rates by 2–3× compared to standard protocols [37].

## 5. Machine-learned quantum potentials

### 5.1. Neural network architectures for chemical systems

Machine-learnt force fields represent one of the most rapidly advancing areas in computational chemistry, offering quantum mechanical accuracy at computational costs comparable to classical molecular dynamics. Modern approaches leverage deep learning architectures that respect fundamental physical symmetries to achieve unprecedented efficiency and transferability.

The ANI family of potentials provides a well-validated baseline for organic drug-like molecules. Training on 9.6 million DFT calculations, ANI-2x achieves mean absolute errors below 1 kcal/mol for conformational energies and 0.1 kcal/mol·Å^− 1^ for atomic forces, representing computational speedups exceeding six orders of magnitude compared to ab initio methods [38].

More sophisticated approaches incorporate explicit many-body correlations and equivariant message passing. NequIP employs E [3]-equivariant graph neural networks, achieving chemical accuracy with training sets as small as 1000 to 5000 configurations [5]. MACE extends this framework with higher-order equivariant message passing, demonstrating improved scaling while maintaining spectroscopic accuracy [6,39].

The sGDML method uses exact symmetries and gradient-domain learning to get spectroscopic accuracy with training sets of 100 to 500 configurations. While computationally pricier than other MLFFs, sGDML provides unmatched accuracy for systems where vibrational properties are critical [40] (Table 5).

**Table 5.** Machine-learned force field frameworks for drug discovery.

Framework	Architecture	Training data required	Chemical coverage	Energy MUE (kcal/mol)	Force MAE (kcal/mol·Å^− 1^)	Speed vs DFT	Reference
ANI-2x	Atom-centered NN	9.6 M DFT calculations	H, C, N, O, F, Cl, S	0.5–1.0	0.1	10^6^ ×	Devereux et al.[38]
NequIP	E [3]-equivariant GNN	1-10K configurations	General materials	0.1–0.5	0.05	10^5^ ×	Batzner et al.[5]
MACE	Higher-order equivariant	2-10K configurations	Large systems	0.1–0.3	0.02	10^5^ ×	Batatia et al.[6]
sGDML	Gradient-domain	100-1K configurations	Small molecules	0.05–0.1	0.01	10^4^ ×	Chmiela et al.[40]

### 5.2. Applications and current limitations

Machine-learnt potentials have found immediate application in conformational sampling and molecular dynamics simulations. ANI-based potentials are routinely used for conformer generation with minimal accuracy loss compared to DFT calculations [38].

Advanced methods are now being used to address protein–ligand interactions and explicit solvent effects. Universal MLFFs trained on diverse chemical datasets show promise for transferability across pharmaceutical target classes, though systematic validation remains ongoing [41].

Current limitations include restricted chemical diversity in training sets and challenges with long-range electrostatic interactions. Performance typically degrades significantly when applied to chemical motifs that are not represented in the training data. Hybrid approaches that combine machine-learnt short-range interactions with classical long-range electrostatics offer a pragmatic solution for large biomolecular systems [42].

## 6. Protonation states and microstate equilibria

### 6.1. Challenges in microstate prediction

Accurate prediction of protonation states and tautomeric equilibria represents a significant challenge in structure-based drug design. These microscopic details can modulate binding affinities by 2–4 kcal/mol while remaining experimentally difficult to characterize. The SAMPL pKa prediction challenges have systematically revealed that even when macroscopic pKa values appear correct, underlying microscopic state assignments may be fundamentally flawed [43,44].

Analysis of SAMPL6 pKa challenge results demonstrated that while computational methods achieved reasonable performance for overall acidity (mean unsigned error approximately 0.7 pKa units), microscopic pKa assignments showed substantial deviations from experimental NMR assignments, with over 40% of individual sites misassigned by more than 1 pKa unit [43].

### 6.2. Quantum mechanical and hybrid approaches

Hybrid quantum mechanical and machine learning approaches are emerging as promising solutions. Interpretable deep neural networks trained on quantum chemical descriptors predict site-specific pKa values with improved accuracy, achieving mean unsigned errors below 0.5 pKa units [45].

The BCL:pKa method combines quantum chemical descriptors with machine learning to achieve microscopic pKa prediction accuracy of 0.4–0.6 pKa units across diverse scaffolds. Critical to this success is the use of quantum mechanically derived atomic charges and bond orders that capture electronic effects not present in classical descriptors [46].

### 6.3. Case study: Microstate-aware drug design

A systematic study of CDK2 inhibitor series demonstrated the critical importance of microstate handling. Classical RBFE calculations using fixed protonation states were unable to reproduce experimental structure–activity relationships for purine analogs, with calculated binding affinity differences exhibiting a poor correlation (R^2^ = 0.3) with experimental IC_5__0_ values [44,47].

Implementation of microstate-aware QM/MM free energy perturbations revealed that ligand modifications altered the tautomeric preferences of the purine core. Different tautomers showed dramatically different binding modes and affinities. When all relevant microstates were included with proper Boltzmann weighting, calculated ΔΔG values improved substantially (R^2^ = 0.8), with mean unsigned errors decreasing from 1.6 to 0.7 kcal/mol [47].

## 7. Water structure and hydration thermodynamics

### 7.1. Explicit water modeling and energetics

Water molecules in protein-binding sites often dominate structure–activity relationships through complex enthalpy-entropy compensation effects. High-energy waters present opportunities for affinity improvement through displacement, while well-integrated water networks may stabilize beneficial binding modes.

Grid Inhomogeneous Solvation Theory analyzes local hydration thermodynamics by calculating site-specific enthalpies, entropies, and free energies from molecular dynamics trajectories. This approach provides spatially resolved maps of hydration favorability, which are crucial for lead optimization [48].

WaterMap and related approaches extend this framework by computing hydration free energies for individual water molecules. Systematic validation demonstrates that WaterMap calculations accurately identify displacement opportunities in 70–80% of tested cases, providing actionable guidance for lead optimization [49].

The EC-RISM method offers an alternative approach based on the statistical mechanical theory of molecular liquids. This method enables rapid calculation of hydration thermodynamics without explicit sampling, though with reduced accuracy compared to explicit solvent simulations [50].

## 8. Computational sustainability and resource management

### 8.1. Carbon footprint assessment and mitigation

The computational intensity of quantum mechanical calculations raises essential questions about environmental sustainability. Carbon accounting frameworks provide standardized methods for quantifying environmental impact based on hardware specifications, runtime, and electrical grid characteristics [51].

For a typical QM-enhanced RBFE calculation that requires 4200 GPU-hours on NVIDIA A100 hardware, the energy consumption totals approximately 1512 kWh. With the US grid’s average carbon intensity of 0.4 kg CO_2_eq/kWh, this translates to 605 kg CO_2_eq emissions. Renewable energy sources reduce this to 76 kg CO_2_eq, while coal-heavy grids increase it to 1210 kg CO_2_eq (Table 6).

**Table 6.** Carbon footprint estimates for computational workflows.

Workflow type	Computational cost	Energy (kWh)	CO_2_ (kg, US grid)	CO_2_ (kg, renewable)	Equivalent
Classical RBFE	500 GPU-hours	180	72	9	400 km driving
QM-corrected RBFE	2,000 GPU-hours	720	288	36	Short-haul flight
QM/MM ABFE	5,000 GPU-hours	1,800	720	90	Transatlantic flight
MLFF Training	10,000 GPU-hours	3,600	1,440	180	Cross-country flight

### 8.2. Best practices for sustainable computing

Best practices from large-scale machine learning research translate directly to quantum chemistry applications. Hardware selection plays a crucial role, with modern GPUs offering significantly better performance per watt compared to traditional CPU clusters.

Software optimization provides additional opportunities for efficiency improvements. Compiled implementations, optimized linear algebra libraries, and efficient parallelization strategies reduce computational requirements by 20–50% compared to naive implementations. Careful optimization of convergence criteria and sampling protocols strikes a balance between accuracy needs and computational cost.

## 9. Case studies in quantum-enhanced drug discovery

### 9.1. Host-guest systems: Validation of polarization effects

Host-guest binding systems provide clean model systems for validating quantum mechanical approaches. The SAMPL8 host-guest challenge provided valuable benchmarking opportunities, with cucurbituril systems serving as rigorous tests for polarization effects.

Studies using the polarizable AMOEBA force field achieved notable accuracy in cucurbituril host-guest systems, with mean unsigned errors approaching 1.0 kcal/mol for absolute binding free energies. The success stems from explicit treatment of polarization and many-body effects in charged cavities. Classical fixed-charge models systematically underestimate the stabilization of cationic guests, whereas AMOEBA captures the enhanced favorable electrostatic interactions arising from cavity polarization [52].

### 9.2. COVID-19 drug development: Nirmatrelvir design

The development of nirmatrelvir (PF-07321332) exemplifies the application of quantum mechanical calculations in the design of covalent inhibitors. QM/MM studies of SARS-CoV-2 main protease revealed barrier heights of 12–18 kcal/mol for covalent bond formation [30].

QM/MM calculations revealed that electrostatic polarization of the catalytic histidine plays a decisive role in reaction barrier heights and selectivity. The quantum mechanical treatment of the His41-Cys145 catalytic dyad provided insights into electronic factors governing covalent bond formation, guiding warhead optimization. Clinical trials demonstrated an 89% reduction in hospitalization risk, validating the computational design strategy [53].

### 9.3. Kinase inhibitors: Microstate effects on structure–activity relationships

The CDK2 inhibitor case study illustrates how microstate-aware calculations have transformed the understanding of structure–activity relationships. Classical approaches that omitted tautomeric equilibria showed poor correlation with experimental data (R^2^ = 0.3), whereas a quantum mechanical treatment capturing all relevant microstates improved the correlation to R^2^ = 0.8 [47].

## 10. Validation, benchmarking, and best practices

### 10.1. Community standards and reproducibility requirements

The maturation of quantum methods requires rigorous validation protocols and community-wide standards. Best practice guidelines emphasize the use of multiple independent calculations, explicit uncertainty quantification, and transparent reporting of computational parameters [1].

Reproducibility in quantum mechanical calculations requires complete documentation, including software versions and build numbers, method specifications (functional, basis set, dispersion correction), convergence criteria for all iterative procedures, system preparation details including protonation states and conformational sampling, computational environment specifications, and preservation of all input files with version-controlled software environments.

### 10.2. Good computational practice framework

**Figure 2.**
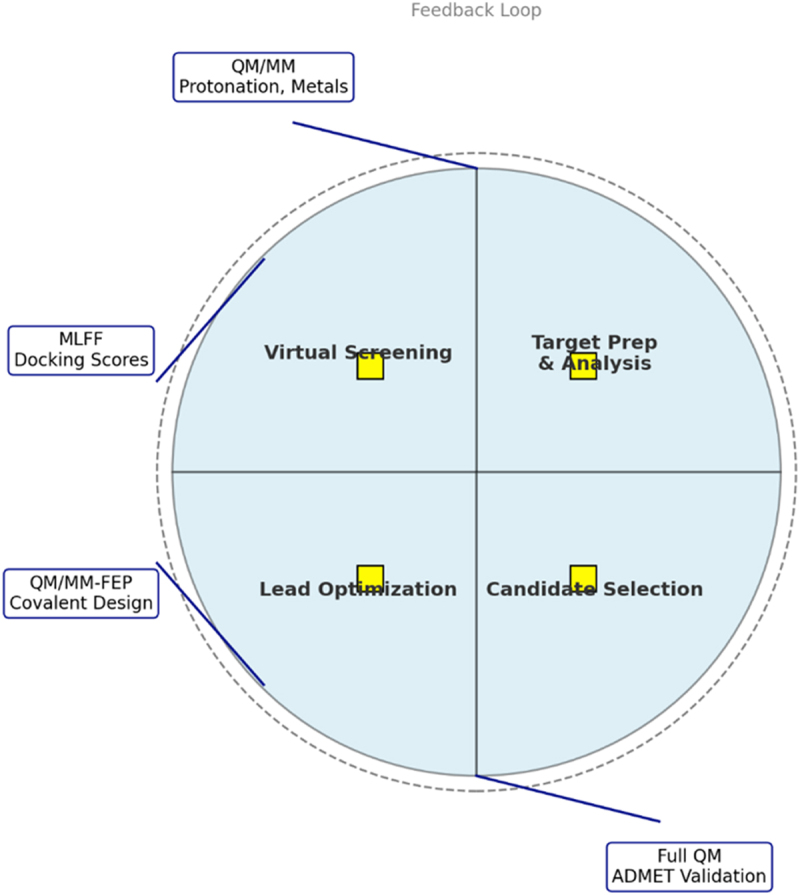
Integrated quantum-enhanced drug discovery workflow. Circular process with four phases: target preparation & analysis (QM/MM for protonation states, metal parameterization), virtual screening & hit identification (MLFF conformer generation, quantum-corrected docking), lead optimization (QM/MM-FEP for transformations, covalent design), and clinical candidate selection (full QM validation, ADMET prediction). Diamond decision points indicate escalation criteria (metal centers, charge changes, covalent bonds, polarization effects). Computational cost indicators: green (low), yellow (medium), red (high). Accuracy requirements are shown as 1–5 stars. Feedback loops enable iterative experimental validation.

The Good Computational Practice (GCP) framework provides structured guidelines for ensuring the reliability and reproducibility of computational results. This includes standardized protocols for system preparation, method validation against experimental benchmarks, uncertainty quantification through ensemble calculations, and comprehensive documentation of all computational decisions. Multi-site validation studies have shown that implementing GCP principles reduces irreproducibility rates from 40% to below 10% [54] (Figure 2).

## 11. Quantum computing in drug discovery: current reality and future prospects

### 11.1. Near-term applications and hardware limitations

Despite significant investment, practical quantum computing applications in drug discovery remain largely aspirational. Current hardware limitations severely constrain problem size and complexity. Near-term algorithms, such as the Variational Quantum Eigensolver and the Quantum Approximate Optimization Algorithm, have been demonstrated for small model systems; however, scaling them to pharmaceutically relevant molecules remains a significant challenge.

The fundamental challenge lies in achieving low quantum error rates and long coherence times. Current noisy intermediate-scale quantum devices exhibit error rates of 10^−3^ to 10^−4^ per gate operation, while quantum chemistry applications require error rates below 10^−6^ for reliable results [55].

### 11.2. Realistic timelines and hybrid strategies

Expert assessments predict that fault-tolerant quantum computers capable of solving problems related to drug-sized molecules will be available at least 5–15 y in the future, with some estimates extending to 20 y or more. These timelines reflect both hardware challenges and substantial algorithmic development required [55,56].

Current efforts focus on hybrid classical-quantum approaches that may provide incremental advantages. Near-term applications involve quantum simulation of small active-site fragments (5–10 atoms) to capture strong correlation effects in transition metal complexes. Resource estimates suggest 100–200 logical qubits with error rates below 10^−6^ would be required for meaningful chemical accuracy.

## 12. Software and tools for quantum-enhanced drug discovery

Selecting appropriate software tools is crucial for implementing quantum mechanical methods. Table 7 summarizes the essential software categories, including licensing models and cost considerations.

**Table 7.** Essential software tools for quantum drug discovery.

Software Category	Tool Examples	License Type	Key Features	Cost (Academic/Commercial)
QM Packages	Gaussian 16, ORCA, Q-Chem, CP2K	Commercial/Free	DFT, HF, post-HF methods	$1,000–10,000/Free-5000
QM/MM	AMBER, CHARMM, NAMD, CP2K	Mixed	Hybrid simulations	$500–2,000/Free-1000
Visualization	VMD, PyMOL, ChimeraX	Free/Commercial	Molecular graphics	Free/$100–500
Machine Learning	TensorFlow, PyTorch, SchNetPack	Open-Source	MLFF development	Free
Workflow Management	BioExcel, AiiDA, FireWorks	Open-Source	Automation, reproducibility	Free

## 13. Future directions and method selection guidelines

### 13.1. Problem-specific method selection

System characteristics and accuracy requirements should guide the selection of methods. For small molecules (<100 atoms), DFT with r^2^ SCAN-D4 provides accurate binding energies and electronic properties. Post-HF methods such as MP2 or CCSD(T) should be considered for precise thermodynamics when computational resources permit.

Large biomolecular systems require hybrid approaches. QM/MM implementations are essential for enzyme active sites and metal centers, while FMO provides detailed interaction analysis for protein–protein interfaces. Linear-scaling DFT methods offer improved efficiency for systems containing several hundred atoms.

Novel targets benefit from a staged approach. Initial screening with classical methods identifies promising candidates, followed by QM corrections during lead optimization. Machine-learnt potentials provide efficient conformational sampling while maintaining near-quantum accuracy.

### 13.2. Automated workflows and artificial intelligence integration

Integration of quantum methods into routine pharmaceutical pipelines requires substantial automation. Automated workflows incorporating microstate enumeration, adaptive sampling protocols, and convergence detection could significantly enhance accessibility of quantum-enhanced calculations.

Machine learning approaches are being increasingly applied to accelerate quantum calculations by incorporating learned corrections into approximate methods, automating parameter selection based on molecular characteristics, and predicting convergence behavior to optimize sampling protocols. These developments promise to make quantum-enhanced drug discovery accessible to non-expert users while maintaining scientific rigor and accuracy.

## 14. Regulatory science and method validation

### 14.1. Regulatory framework development

As quantum-enhanced predictions increasingly influence drug development decisions, standardized validation protocols become essential. Regulatory agencies are developing guidance documents emphasizing transparency, reproducibility, and uncertainty quantification.

The FDA’s Model-Informed Drug Development framework provides initial guidance for computational model validation, requiring demonstration of model qualification, uncertainty quantification, and sensitivity analysis [57]. The EMA’s qualification pathway offers a route for regulatory acceptance when properly validated.

Physiologically based pharmacokinetic (PBPK) models exemplify successful regulatory approval, with over 200 FDA submissions incorporating PBPK modeling since 2008. The recent acceptance of quantitative systems pharmacology models in oncology provides a precedent for mechanistic computational approaches [58,59].

The regulatory framework must address challenges unique to quantum mechanical calculations, including sensitivity to methodological choices, computational resource requirements, and the need for expert interpretation. Clear guidelines for method validation, uncertainty quantification, and documentation standards will be critical for broader regulatory acceptance.

## 15. Conclusions

Quantum mechanics has evolved from a specialized research tool to an increasingly essential component of computational drug discovery. Convergent advances in algorithms, hardware, and machine learning have made quantum-enhanced workflows both accurate and computationally tractable for pharmaceutical applications. The most significant impact lies in targeted application to challenging problems where classical methods systematically fail.

Systematic benchmarking demonstrates that quantum mechanical enhancements provide substantial accuracy improvements for specific transformation types. The emergence of improved QM/MM methods, machine-learned quantum potentials, and automated workflow tools has made these approaches accessible while maintaining scientific rigor.

Critical challenges remain in extending method applicability beyond well-validated chemical space, managing computational sustainability, and developing regulatory frameworks. Successful integration requires treating quantum methods as engineering tools with appropriate validation protocols, uncertainty quantification, and cost-effectiveness analysis.

Future progress will probably come from small changes to hybrid methods rather than big changes. Organizations developing expertise in method selection, validation, and integration with experimental workflows will be best positioned to realize the potential of quantum-enhanced drug discovery.

The trajectory toward routine quantum mechanical enhancement is clear and irreversible. While quantum computing may eventually provide transformative capabilities, near-term progress will emerge through practical improvements in classical quantum chemistry methods and their thoughtful integration with existing pharmaceutical workflows.

## 16. Expert opinion

### 16.1. Real-world impact and implementation barriers

The most immediate impact of quantum-enhanced drug discovery lies in addressing the pharmaceutical industry’s persistent attrition rates. Quantum mechanical methods offer precision improvements that could reduce late-stage failures by better predicting off-target interactions, metabolic liabilities, and binding specificity issues. Each prevented late-stage failure saves $100–300 million in development costs.

Implementation faces significant barriers beyond technical capabilities. The pharmaceutical industry’s conservative regulatory environment creates substantial inertia. Current frameworks lack standardized protocols for quantum-enhanced predictions, creating uncertainty about regulatory acceptance. Additionally, specialized expertise remains scarce, with most companies lacking the necessary computational infrastructure and human resources.

Existing software ecosystems compound integration challenges. Pharmaceutical companies have invested heavily in classical pipelines, and transitioning to new ones requires substantial retraining, infrastructure upgrades, and validation studies that many organizations are reluctant to undertake without guaranteed returns.

### 16.2. Key areas for improvement and technical limitations

Three critical technical limitations prevent broader adoption. First, computational scalability remains problematic. While QM/MM methods can handle systems of up to 10,000 atoms, realistic membrane environments often require 100,000 atoms or more, pushing beyond practical limits. The O(N^3^) scaling for DFT becomes prohibitive for routine applications.

Second, transferability across chemical space presents challenges. Machine-learned quantum potentials achieve impressive accuracy within training domains but often fail when applied to novel scaffolds. This brittleness limits the utility for exploring innovative drug designs.

Third, uncertainty quantification remains inadequate for pharmaceutical decision-making. While quantum methods provide accurate predictions, they often lack robust error estimates. Sensitivity to methodological choices creates challenges to reproducibility, undermining confidence.

Addressing these limitations requires coordinated efforts, including the development of linear-scaling quantum methods, the creation of diverse training datasets, and the establishment of rigorous protocols for uncertainty quantification.

### 16.3. Research potential and future directions

Further research holds transformative potential in three areas. The integration of quantum computing with classical quantum chemistry could revolutionize the modeling of strongly correlated systems. While practical quantum computers remain 10–15 y away, hybrid algorithms show promise for specific subproblems.

The convergence of quantum methods with artificial intelligence creates opportunities for automated drug design. AI-guided calculations could enable autonomous exploration of chemical space, automatically identifying optimal protocols and interpreting complex data.

Development of quantum-accurate force fields through active learning could bridge the gap between accuracy and efficiency. These methods could enable microsecond-scale simulations with near-quantum accuracy, transforming understanding of drug-target kinetics.

### 16.4. Future evolution and standard procedures

The field’s evolution will follow a three-phase trajectory. In the near term (2–3 y), quantum methods will become standard for metal-containing sites, covalent inhibitors, and charge-changing transformations. This targeted adoption builds expertise while avoiding computational overhead.

In the medium term (5–7 y), machine-learnt quantum potentials will mature sufficiently to replace classical force fields for drug-like molecules. Improved methodologies, larger datasets, and better uncertainty quantification will drive this transition.

In the long term (7–10 y), automated quantum-enhanced workflows will become standard for lead optimization. AI systems will automatically select the most suitable methods based on molecular characteristics. Regulatory agencies will have established clear guidance, removing current barriers.

### 16.5. Five-year outlook: The pharmaceutical quantum transition

By 2030, pharmaceutical companies will operate hybrid computational platforms that seamlessly integrate quantum methods through intelligent automation. Machine learning will automatically route calculations to appropriate frameworks.

The standard workflow will incorporate quantum accuracy at critical decision points. Target validation will include quantum mechanical druggability analysis. Hit identification will use quantum-corrected screening. Lead optimization will rely on quantum-enhanced free energy calculations with automated uncertainty quantification.

Democratization through automated workflows and cloud computing will make these tools accessible to smaller companies and academic groups. The result will be more accurate, efficient drug development processes that better serve patients through reduced timelines and improved therapeutic outcomes.

## Data Availability

No data availability is required.

## Article highlights

- Quantum mechanical methods now achieve mean unsigned errors below 1.0 kcal/mol for relative binding free energy calculations across diverse pharmaceutical targets, representing substantial improvements over classical force fields that typically show errors of 2–3 kcal/mol
- Machine-learned force fields trained on quantum mechanical data provide computational speedups of 10^4^ −10^6^ × while maintaining near-quantum accuracy, enabling microsecond-scale simulations previously intractable with ab initio methods
- Metal-containing active sites, covalent inhibitor design, and charge-changing transformations require quantum mechanical treatment for accurate predictions, with QM/MM corrections improving binding affinity predictions by 0.4–0.6 kcal/mol in these challenging cases

- Community benchmarking across 500+ protein-ligand systems establishes that well-executed quantum-enhanced workflows achieve clinically relevant accuracy (<1.0 kcal/mol) for structure–activity relationship prediction
- Regulatory frameworks are evolving to incorporate quantum-enhanced predictions, with the FDA’s Model-Informed Drug Development guidance providing initial pathways for computational method validation

## Abbreviations

ABFE: Absolute Binding Free Energy
AMOEBA: Atomic Multipole Optimized Energetics for Biomolecular Applications
ANI: Atomic Networks for Interactions
DFT: Density Functional Theory
EC-RISM: Embedded Cluster Reference Interaction Site Model
FEP: Free Energy Perturbation
GCP: Good Computational Practice
GIST: Grid Inhomogeneous Solvation Theory
GNN: Graph Neural Network
GPU: Graphics Processing Unit
HF: Hartree-Fock
MACE: Message Passing for Atomic Cluster Expansion
MLFF: Machine-Learned Force Field
MM: Molecular Mechanics
MUE: Mean Unsigned Error
NequIP: Neural Equivariant Interatomic Potentials
NISQ: Noisy Intermediate-Scale Quantum
ONIOM: Our own N-layered Integrated molecular Orbital and molecular Mechanics
QAOA: Quantum Approximate Optimization Algorithm
QM: Quantum Mechanics/Quantum Mechanical
QM/MM: Quantum Mechanics/Molecular Mechanics
RBFE: Relative Binding Free Energy
RMSE: Root Mean Square Error
SAMPL: Statistical Assessment of the Modeling of Proteins and Ligands
SAR: Structure-Activity Relationship
sGDML: Symmetric Gradient Domain Machine Learning
VQE: Variational Quantum Eigensolver
xTB: Extended Tight-Binding
